# Unenhanced magnetic resonance imaging in the risk stratification of indeterminate adnexal masses: a viable solution in the face of clinical limitations?

**DOI:** 10.1590/0100-3984.2025.58.e1-en

**Published:** 2025-08-01

**Authors:** Luis Ronan Marquez Ferreira de Souza, Cecilia Vidal de Souza Torres

**Affiliations:** 1 Universidade Federal do Triângulo Mineiro (UFTM), Uberaba, MG, Brazil; 2 Hospital das Clínicas da Faculdade de Medicina de Ribeirão Preto da Universidade de São Paulo (HCFMRP-USP), Ribeirão Preto, SP, Brazil

The evaluation of adnexal masses continues to be a relevant clinical challenge, especially when there is a possibility of malignancy. Ovarian cancer, which remains the most lethal gynecological malignancy, often presents nonspecific symptoms or is asymptomatic in the early stages, resulting in delayed diagnosis and a poorer prognosis^(1)^. In this context, accurate stratification of malignancy risk is essential to guide management, avoid unnecessary surgeries, and prioritize patients at higher risk. Transvaginal ultrasound, as a first-line screening method, is highly sensitive for characterizing many ovarian lesions and is often the only method necessary. However, up to 25% of masses are indeterminate on ultrasound, and magnetic resonance imaging (MRI) emerges as a fundamental tool in such cases, especially when applied with structured protocols supported by risk classification systems such as Ovarian-Adnexal Reporting and Data System (O-RADS) MRI scoring^(2,3)^, which increase specificity. In addition, the use of percutaneous biopsy for such lesions is often ruled out as an option because of the associated risk of tumor cell dissemination and the possibility of a sampling error. However, the use of intravenous contrast is a technical requirement of O-RADS MRI scoring^(4)^, which limits its applicability in patients with contraindications to the use of gadolinium or in resource-constrained settings.

The study conducted by Moradi et al.^(5)^ and recently published in Radiologia Brasileira makes a significant contribution. The authors explored the diagnostic feasibility of an unenhanced MRI protocol for the evaluation of adnexal masses that were deemed indeterminate on ultrasound. In a multicenter retrospective study that evaluated 336 masses in 243 patients, the authors demonstrated a sensitivity of 97.7%, specificity of 86.4%, and accuracy of 93.8% for the detection of malignancy, with an area under the receiver operating characteristic curve of 0.944. The proposed scoring system is based on morphological and functional criteria obtained from T1-, T2-, and diffusion-weighted sequences, with subjective assessment of true restriction on the apparent diffusion coefficient map. The authors found high interobserver agreement on the classification of adnexal lesions (kappa = 0.9). However, it is important to highlight the limitations of the study, such as the relatively small number of borderline cases, the potential bias of interpretation by radiologists experienced in gynecological imaging (which could compromise reproducibility in settings with less experienced evaluators), the subjective evaluation of diffusion findings, and the absence of quantitative criteria, all of which limit the reproducibility of the scoring system.

The findings of Moradi et al.^(5)^, although preliminary, spark an interesting discussion and have major clinical implications. The literature points to the growing need for prospective multicenter studies that validate unenhanced MRI protocols in contexts in which contrast cannot be used. This approach is especially relevant for patients with contraindications to the use of gadolinium and represents a strategy to optimize examination time and resource allocation at facilities where there is a high demand for MRI. Similarly, the absence of the uterus due to hysterectomy can impact the application of O-RADS MRI scoring, because the system uses the comparison of the lesion enhancement with that of the myometrium as a reference for evaluating the enhancement pattern in contrast-enhanced examinations. Without this anatomical reference, the low-risk curve can be recognized by its morphology (progressive enhancement without a plateau) but it is not possible to distinguish between the intermediateand high-risk curves. This is another situation in which an alternative to a contrast-enhanced evaluation would be interesting.

It is necessary to exercise caution when interpreting borderline tumors and masses with mixed characteristics, the risk of which can be underestimated when dynamic contrast-enhanced sequences are not acquired. The Moradi et al.^(5)^ study not only underscores the relevance of diffusion in pelvic evaluation but also highlights the fact that the interpretative approach needs to be contextualized with other imaging findings and clinical history. The utility of ultrasound should not be underestimated, because it can be used as a complement to an unenhanced MRI protocol in order to better characterize adnexal lesions. The future of adnexal mass evaluation seems to be moving toward individualized protocols that consider not only the ideal technique but also the profile of the patient, local expertise, and available resources.

To validate the proposed scoring system, there is a need for prospective multicenter studies in which it is applied by radiologists with different levels of experience. In addition, the incorporation of quantitative tools and artificial intelligence could increase the accuracy and standardization of the unenhanced imaging approach.

Given the constant evolution of gynecological imaging, the work of Moradi et al.^(5)^ adds to international efforts to make diagnostic imaging more accessible, safe, and patient-centered, without sacrificing accuracy. Their findings can inform attempts to find alternatives to the traditional standards established in the literature.
